# A Systematic Review of the Association of Internet Gaming Disorder and Excessive Social Media Use With Psychiatric Comorbidities in Children and Adolescents: Is It a Curse or a Blessing?

**DOI:** 10.7759/cureus.43835

**Published:** 2023-08-21

**Authors:** Sally Ghali, Shadin Afifi, Vineet Suryadevara, Yaman Habab, Alana Hutcheson, Binay K Panjiyar, Gershon G Davydov, Hiba Nashat, Tuheen Sankar Nath

**Affiliations:** 1 Psychiatry/Neuroscience, California Institute of Behavioral Neurosciences & Psychology, Fairfield, USA; 2 Internal Medicine, California Institute of Behavioral Neurosciences & Psychology, Fairfield, USA; 3 Cardiology, Harvard Medical School, Boston, USA; 4 Nephrology, California Institute of Behavioral Neurosciences & Psychology, Fairfield, USA; 5 Internal Medicine, Soroka University Medical Center, Be'er Sheva, ISR; 6 Surgical Oncology, Tata Medical Centre, Kolkata, IND

**Keywords:** mental disorders, mental illnesses, digital media use, social media use, internet addiction disorder, internet gaming disorder

## Abstract

Internet gaming and social media usage (SMU), particularly among children and teenagers, have witnessed a remarkable surge over the past decade. However, it remains uncertain whether this widespread usage has a positive or negative impact. The primary objective of this systematic review was to investigate the diverse effects of excessive video game playing and extensive SMU, both favorable and detrimental, on the psychological and mental well-being of children and adolescents. To assess the influence of internet gaming disorder (IGD) and disordered SMU on the mental health of children aged 6-12 years and adolescents aged 13-18 years, we conducted a systematic review of 20 studies on the subject. These studies utilized a substantial sample size of 48,652 participants, encompassing online and in-person questionnaires administered to children, teenagers, and their parents in educational institutions, healthcare facilities, and online platforms. Our findings suggest that multiple factors contribute to the intricate relationship between SMU, video game playing, and mental health outcomes. The majority of research indicates that excessive gaming or SMU among children and teenagers leads to adverse consequences on their mental well-being.

Furthermore, certain studies have even reported fatal consequences, while others have identified a worsening of preexisting mental health issues. A few studies have explored the potential positive impacts of SMU and gaming on individuals and society at large. In light of this, we have concluded that it is inappropriate to categorize internet gaming and SMU as solely beneficial or detrimental without considering the broader context and the interplay of various factors.

## Introduction and background

The domain of mental health literacy, which encompasses an individual's knowledge and understanding of their own mental health concerns, has witnessed remarkable advancements in recent decades [1]. It is crucial to foster the development of health literacy within any given population. This can effectively empower individuals to recognize and address the challenges they face while promoting the adoption of preventive measures and avoidance of risky behaviors that may exacerbate their psychological or physical well-being. Additionally, health literacy ensures that individuals are aware of the availability of professional assistance and encourages the positive mindset required to seek help when necessary. Notably, as mental health concerns continue to gain global recognition, particular attention is directed toward children and adolescents. Before reaching adolescence, mental health disorders constitute the most prevalent form of illness among young individuals [2]. Surprisingly, approximately 50% of all mental illnesses manifest symptoms by the age of 14 years, and disturbances in mood regulation can even emerge as early as 11 years of age [2].

Over the past two decades, the internet has profoundly transformed people's lives worldwide [3]. Among all age groups, children and teenagers, the most frequent users, are particularly affected by its influence [4]. The utilization of the internet by students poses a significant concern in all elementary schools. To address this issue and mitigate excessive internet use, some educational institutions have implemented interventions such as "No Media Day" [5]. Research has indicated a prevalence of internet addiction among students in the United States ranging from 5% to 25%, underscoring the need for thorough investigation and recognition of this phenomenon [6]. It is commonly assumed that children who spend extensive time online tend to become increasingly detached from reality [7]. Consequently, we were intrigued enough to explore whether the prevailing excessive use of the internet is genuinely detrimental to the mental health of children and adolescents, or, if, when utilized appropriately, it can potentially yield positive effects on their social and cognitive development.

**Figure 1:**
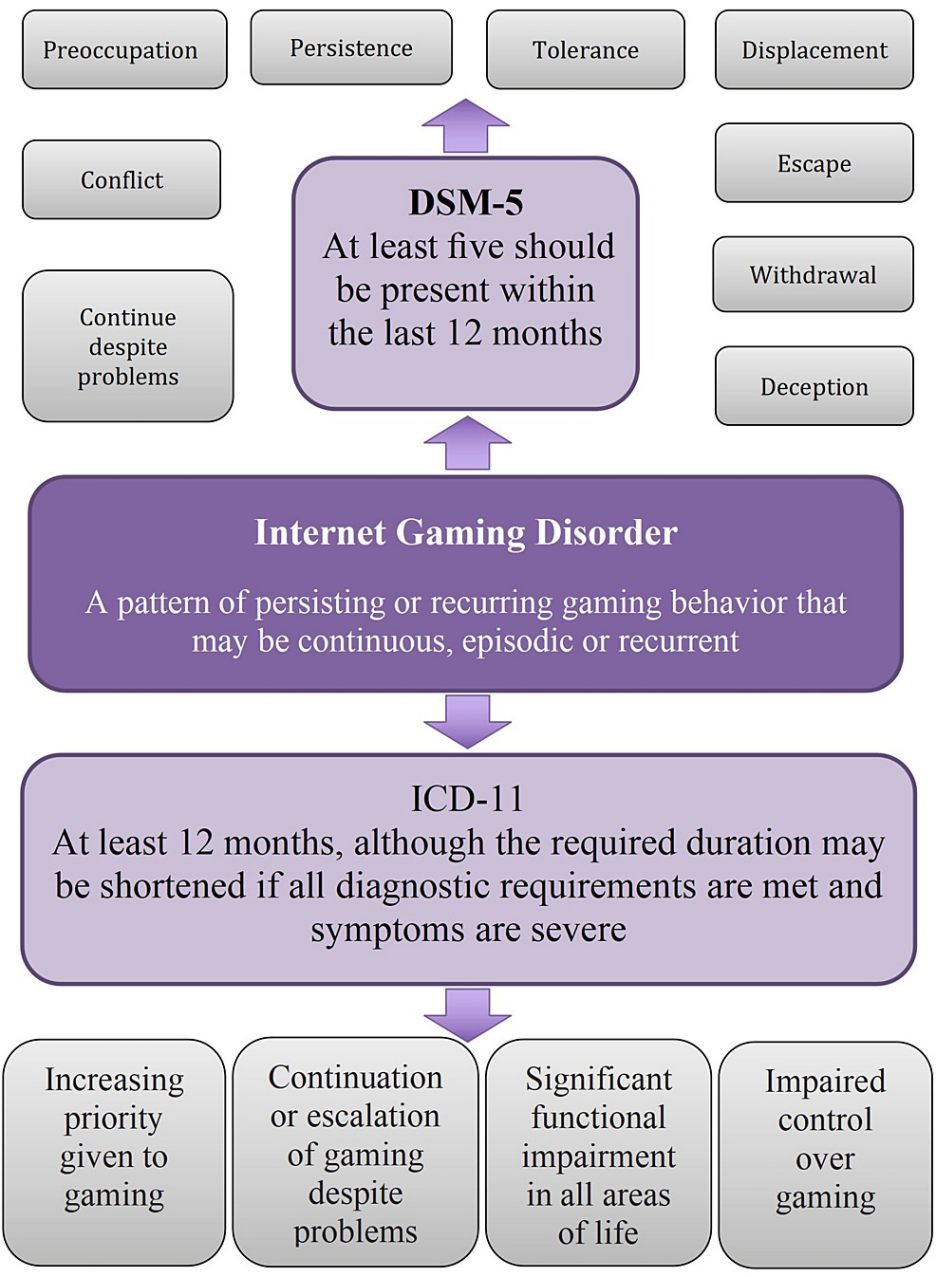
Internet gaming disorder diagnostic criteria in DSM-5 and ICD-11 DSM-5: Diagnostic and Statistical Manual of Mental Disorders, Fifth Edition; ICD-11: International Classification of Diseases 11th Revision Image credits: Sally Ghali

**Figure 2:**
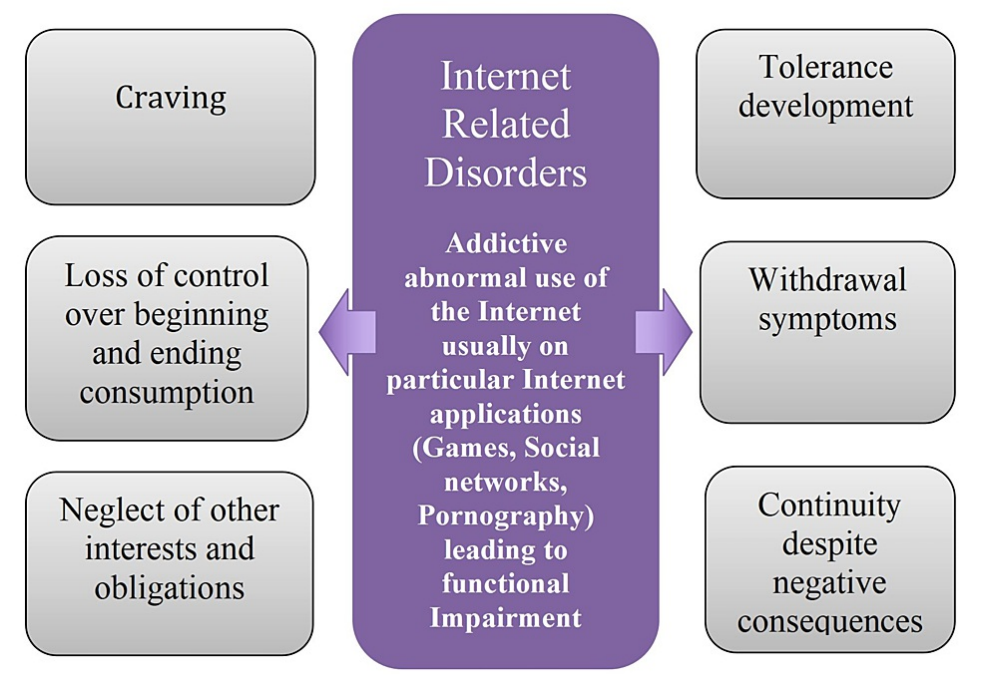
The patterns of internet-related disorders Image credits: Sally Ghali

A subject of ongoing debate revolves around the terminology used to describe the issue at hand. Various terms, such as internet gaming disorder (IGD), internet addiction disorder (IAD), problematic internet use (PIU), and social media usage (SMU), are all synonymous with excessive and problematic behavior exhibited on the internet [8]. Both the Diagnostic and Statistical Manual of Mental Disorders, Fifth Edition (DSM-5) (American Psychiatric Association, 2013) and the International Classification of Diseases 11th Revision (ICD-11) (World Health Organization, 2019) have classified IGD as a mental health condition (Figure 1) [8,9]. In contrast, the concept of addictive-like SMU serves as an umbrella term encompassing addictive-like behaviors across multiple social networking sites (e.g., Facebook, Twitter, Tik-Tok, Instagram) and instant messaging platforms (e.g., WhatsApp, Viber, Snapchat). Currently, it does not hold a distinct position in the DSM-5 or ICD-11. However, it is important to note that a significant number of online activities exhibit similar patterns of behavior (Figure 2). Thus, we aim to comprehensively shed light on all aspects concerning the use of digital media in children and adolescents, including IGD.

## Review

Methodology

In conducting this systematic review, we relied on a range of databases including PubMed, Research Gate, Google Scholar, and the Medical Subject Headings (MeSH) thesaurus. To search specifically on PubMed, we utilized the following MeSH strategy: "Internet Gaming Disorder" OR ("Internet Addiction Disorder/etiology"[Majr] OR "Internet Addiction Disorder/Majr]) AND Mental illnesses OR "Mental Disorders/diagnosis[Majr]". By implementing this MeSH strategy, we aimed to identify relevant articles that specifically addressed the topics of IGD and IAD in relation to mental illnesses and mental disorders. This strategy allowed us to retrieve articles that focused on the etiology, diagnosis, and associated factors of these conditions. Through the utilization of appropriate MeSH terms and logical operators, we sought to ensure the inclusion of articles aligned with our research objectives.

To conduct our literature search, we employed several search terms to capture relevant studies. The search terms utilized include internet gaming disorder, internet addiction disorder, social media use, digital media use, mental illnesses, and mental disorders. To refine our search results, we utilized logical operators such as 'AND', 'OR', and 'NOT' to combine these terms in different ways. By using these operators, we were able to construct search queries that encompassed the relationships between these terms and focused on the specific aspects of interest for our study. This approach allowed us to retrieve a comprehensive range of articles related to IGD, IAD, SMU, digital media use, and their associations with mental illnesses and disorders.

Inclusion and Exclusion Criteria

In our study, we specifically focused on children aged 6-12 years and adolescents aged 13-18 years. We included a total of 18 observational studies and two review articles that met the following inclusion criteria: written in English, published within the last five years, and focusing on our defined age groups. Publications that fell outside of the five-year timeframe, articles that centered on the adult population, and works in languages other than English were excluded from our analysis. By applying these selection criteria, we aimed to ensure the relevance and timeliness of the included studies in addressing our research objectives.

Results

In our systematic review, we included a total of 20 studies. Most of them utilized a combination of online and in-person questionnaires, which were completed by either the participants themselves or their parents. These studies were categorized as observational cross-sectional studies or review articles. The cumulative number of questionnaires employed to assess the effects of IGD and SMU on children aged 6-12 years and adolescents aged 13-18 years amounted to 48,652. Unfortunately, the sample size used in one of the literature reviews was not specified [8]. The majority of the included studies indicated that the population of children and adolescents experienced various psychological effects as a result of excessive internet gaming and social SMU. These effects encompassed conditions such as depression, attention deficit and hyperactivity disorder (ADHD), autism spectrum disorder (ASD), sleep disturbances, decline in school performance, school refusal, low life satisfaction, impaired social cognition and emotion dysregulation, eating disorders, body dissatisfaction, increased risk of sexual victimization, and suicidal ideation and attempts [2,5,7,10-22]. One study specifically highlighted the exacerbation of preexisting mental disorders, linking ADHD to disruptive mood dysregulation disorder (DMDD) in the presence of internet gaming problems [23]. Additionally, another study revealed the potential risks associated with prolonged video game-playing sessions, leading to mortality [24]. Lastly, a few studies reported some positive effects on academic performance, as well as mental and social well-being [9,15,17,25-27]. The summary of the studies used in our review is presented in Table 1.

**Table 1:** Summary of the studies N/A: not applicable

Author and year of publication	Number of patients	Study type	Purpose of the study	Conclusion
Jeon et al., 2022 [1]	169	Cross-sectional observational	To investigate mental health literacy of internet gaming disorder and problematic smartphone use in Korean teenagers based on their capacity to recognize addictions	Internet gaming disorder and problematic smartphone use are little understood by teenagers. There must be educational initiatives to aid in their comprehension
Jeong et al., 2019 [2]	366	Cross-sectional observational	To assess the association between internet gaming disorder and depression	Internet gaming disorder severity and depression levels are bidirectionally correlated
Yamada et al., 2021 [5]	13413	Cross-sectional observational	Examining the incidence and contributing factors to pathologic internet use and dangerous online behavior in schoolchildren	Children in elementary school frequently engage in dangerous behaviors and use the internet harmfully
Fujita et al., 2022 [7]	227	Cross-sectional observational	Assessing daily struggles and improper internet use among teenagers if linked to school refusal	Parent assessments of daily challenges are impacted by problematic internet use, especially throughout the day
Geisel et al., 2021 [8]	N/A	Literature review	Assessing dangers and risks associated with internet use	Internet-related disorders are not yet well understood and the concept of illness and its treatment has to be improved further
van den Eijnden et al., 2018 [9]	538	Cross-sectional observational	To evaluate the effects of teens' engaged and disordered use of games and social media on their psychosocial well-being and academic performance	Teenagers' psychological well-being and academic performance are predicted to decline as a result of the signs of disordered usage of video games and social media
Saatçioğlu et al., 2023 [10]	90	Cross-sectional observational	To assess the ability of individuals with internet addiction, internet addiction plus attention deficit and hyperactivity disorder to regulate their emotions and social behavior	Social cognition and emotion regulation are problems for patients with internet addiction and internet addiction in combination with attention deficit and hyperactivity disorder
Restrepo et al., 2020 [11]	564	Cross-sectional observational	To assess the association between problematic internet use and psychiatric disorders	A positive correlation exists between depression, autism spectrum disorder, attention deficit and hyperactivity disorder, and problematic internet use
Seo et al., 2020 [12]	180	Cross-sectional observational	Understanding how depressive symptoms can act as a mediator in the association between negative childhood experiences and problematic internet use	Depressive symptoms were found to have a strong mediation effect on the association between problematic internet use and negative childhood experiences
Tereshchenko et al., 2021 [13]	4615	Cross-sectional observational	To assess the association between internet addiction and sleep problems	Significant disturbances in sleep among adolescents with internet addiction
Yoon et al., 2021 [14]	4940	Cross-sectional observational	Examining the impact of smartphone addiction on sleep duration while controlling for age and gender	Males slept for longer than females, but there was no gender difference in smartphone addiction. Smartphone addiction, particularly the tolerance sub-factor, considerably impacted sleep length. Sleep duration was strongly affected by age, while gender had a moderating effect
Islam et al., 2020 [15]	1704	Cross-sectional observational	Investigating how playing games and using the internet affect academic achievement	The use of the internet was linked to poorer academic performance. Online gaming, however, was connected to higher reading test results
Phan et al., 2019 [16]	2400	Cross-sectional observational	Examining the connection between life satisfaction and internet gaming disorder symptoms while taking other factors into consideration	In older individuals, internet gaming disorder was substantially linked to lower life satisfaction, and the symptoms may vary by gender and age
Kerr and Kingsbury, 2023 [17]	13600	Cross-sectional observational	To analyze the relationship between the use of different forms of digital media and mental health	There was only one significant correlation between the frequency of digital media use and eating disorders once other factors were adjusted for
Messena and Everri, 2023 [19]	129	Scoping review	To explain the connection between children's wellness and the improper use of digital technology	A lack of cognitive well-being is caused by inappropriate online activity and compulsive digital use
Tamarit et al., 2021 [20]	1763	Cross-sectional observational	Examining how body self-esteem affects the link between internet addiction and risky sexual and erotic activity	Body self-esteem and sexting act as mediators between internet addiction and sexual online victimization. Internet addiction foretells online sexual exploitation
Tzang et al., 2022 [23]	102	Cross-sectional observational	To ascertain whether patients with attention deficit and hyperactivity disorder who have internet gaming disorder can develop into disruptive mood dysregulation disorder	Internet gaming disorder facilitates the development of signs of disruptive mood dysregulation
Kuperczko et al., 2022 [24]	24	Observational	To Assess the association between video games and death	After exceptionally extended gaming sessions, the incidence is quite low and predominantly affects young people
Wichstrøm et al., 2019 [28]	740	Cross-sectional observational	To evaluate the symptoms, comorbidities, and predictors of internet gaming disorder	Inability to identify various correlates and predictions, as well as a weak association between some symptoms and internet gaming disorder
Kim et al., 2022 [29]	3217	Cross-sectional observational	Understanding of the variations in internet gaming addiction and personality traits based on the game genre	Depending on the type of game played, different personality traits and internet gaming addictions exist

In our systematic review, we carefully assessed the quality and potential bias of the included studies. A total of 28 studies were initially chosen for evaluation, and two authors utilized established quality assessment tools [the Newcastle-Ottawa Scale for observational studies and the Scale for the Assessment of Narrative Review Articles (SANRA) for traditional reviews] to review their methodological rigor and overall quality. Following this assessment, 20 articles were identified as meeting the criteria of medium or high-standard quality and subsequently included in the systematic review. This rigorous selection process ensured that only studies of sufficient quality were included in our analysis, while any studies deemed to be subpar were disqualified. By employing these quality assessment measures, we aimed to maintain the reliability and validity of the evidence included in our systematic review. Results for the quality evaluation of each study in our review are shown in Tables 2-3.

**Table 2:** Quality assessment results for observational studies

Newcastle-Ottawa Scale assessment		Representativeness of the sample	Sample size	Non-respondents	Ascertainment of the exposure	Comparability	Assessment of the outcome	Statistical test	Total
Jeon et al., 2022 [1]		1	1	1	0	2	2	1	8
Jeong et al., 2019 [2]		1	1	1	0	2	1	1	7
Yamada et al., 2021 [5]		1	1	1	0	2	2	1	8
Fujita et al., 2022 [7]		1	1	1	0	0	2	1	6
van den Eijnden et al., 2018 [9]		1	1	1	0	2	1	1	7
Saatçioğlu et al., 2023 [10]		1	1	1	1	2	2	1	9
Restrepo et al., 2020 [11]		1	1	1	0	2	1	1	7
Seo et al., 2020 [12]		1	1	1	0	0	2	1	6
Tereshchenko et al., 2021 [13]		1	1	1	2	2	1	1	9
Yoon et al., 2021 [14]		1	1	1	1	2	2	1	9
Islam et al., 2020 [15]		1	1	1	0	2	2	1	8
Phan et al., 2019 [16]		1	1	1	0	2	1	1	7
Kerr and Kingsbury, 2023 [17]		1	1	1	1	2	0	1	7
Tamarit et al., 2021 [20]		1	1	0	1	2	2	1	8
Tzang et al., 2022 [23]		1	1	1	0	2	1	1	7
Kuperczko et al., 2022 [24]		1	1	0	0	2	2	0	6
Wichstrøm et al., 2019 [28]		1	1	1	2	2	1	1	9
Kim et al., 2022 [29]		1	1	1	0	2	2	1	8

**Table 3:** Quality assessment results for review articles SANRA: scale for the assessment of narrative review articles

SANRA scale assessment	Justification of the article’s importance for the readership	Statement of concrete aims/formulation of questions	Description of the literature search	Referencing	Scientific reasoning	Appropriate presentation of data	Total
Messena and Everri, 2023 [19]	1	1	2	1	1	2	8
Geisel et al., 2021 [8]	2	2	2	2	1	2	11

**Figure 3:**
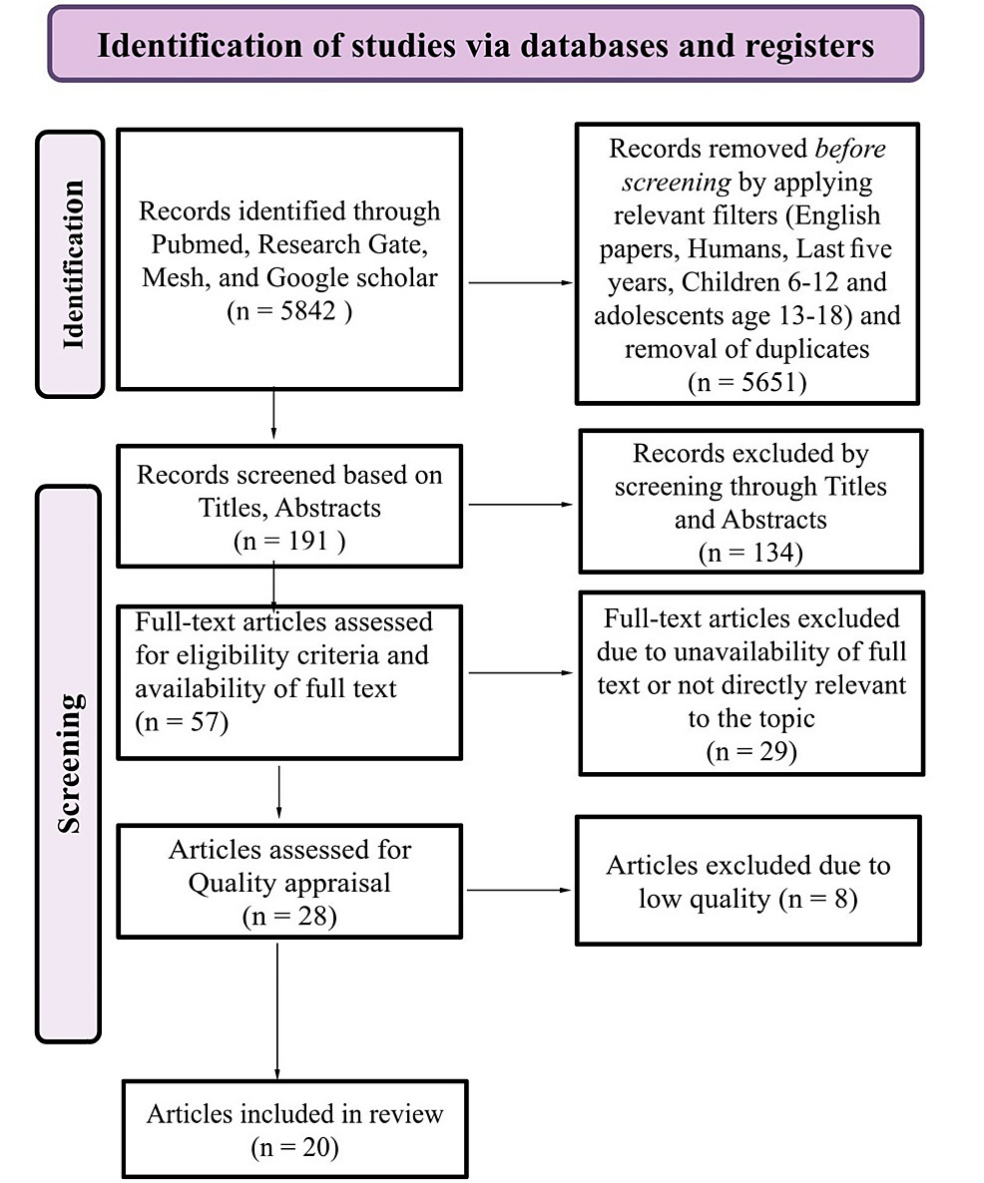
PRISMA flow diagram PRISMA: Preferred Reporting Items for Systematic Reviews and Meta-Analyses; Mesh: Medical subject headings

While conducting this systematic review and in the subsequent derivation of our conclusions, we diligently adhered to the guidelines stipulated by the Preferred Reporting Items for Systematic Reviews and Meta-Analyses (PRISMA) criteria [30]. Our initial search encompassed four databases, yielding a total of 5842 articles. However, through a series of meticulous screening processes, including the acquisition of full-text articles and relevancy to our topic, we refined our selection to include 28 relevant studies. Finally, after careful scrutiny and consideration of the quality assessment results, we included a total of 20 relevant high- or medium-quality studies in our comprehensive systematic review. The PRISMA flow diagram that summarizes our entire selection procedure is shown in Figure 3.

Discussion

Research suggests that IGD and SMU are significant contributors to the increasing prevalence of mental health difficulties among children and teenagers [9,17]. However, the exact determinants of these difficulties are a subject of ongoing debate, including factors such as the amount of time spent online, the specific types of media used, and potential gender disparities. In one of the studies we examined, it was found that certain hidden family characteristics, such as a lack of rules at home or infrequent parent-child interaction, may indirectly contribute to problematic internet use [5]. Addressing these underlying factors and others, if effectively managed, could potentially have a significant impact on the treatment of these conditions. Furthermore, there is a lack of clarity regarding the specific physical, social, and psychological challenges that individuals may encounter as a result of excessive internet gaming and social media use. Understanding and exploring these challenges is crucial to develop targeted interventions and support systems. By delving into these difficulties, our discussion aims to shed light on the multifaceted nature of the issue and highlight the potential areas for intervention and improvement.

Harmful Outcomes

Emotion regulation and social cognition: according to DSM-5 Section III and ICD-11, the diagnosis of IGD requires a minimum of 8-10 hours or more per day, or at least 30 hours per week, of internet use [10]. Similarly, diagnosing internet addiction typically involves using the internet for more than five hours per day and 38.5 hours per week [31].

In a cross-sectional study conducted by Saatçioğlu et al., a group of adolescents (n=90) aged 12-17 years was assessed for emotion regulation (the individual's ability to be aware of their emotions and effectively manage and regulate them) and social cognition abilities (the individual's ability to perceive, interpret, and understand social information, including their own and others' thoughts, feelings, intentions, and behavior). The study included three groups: 30 individuals with IAD, 30 individuals with both IAD and ADHD, and 30 healthy controls [10]. The study found that those with IAD or IAD comorbid with ADHD spent over 40 hours per week online for entertainment purposes, including social media and gaming. As a result, they demonstrated impairments in social cognition and emotion regulation abilities (p<0.001). The effects were even more severe in the group with comorbid ADHD [32]. In contrast, the control group used the internet for an average of 11 hours per week, primarily for educational purposes and completing assignments (p<0.001). The study noted that both genders used the internet for similar reasons, although the difference in the number of hours spent online was small. However, it is worth mentioning that the study's sample size was small, and a larger sample size would have strengthened the validity of the conclusions.

These findings highlight the significant impact of excessive internet use on social cognition and emotion regulation abilities, particularly in individuals with IAD and comorbid ADHD. It also highlights the importance of considering the amount of time spent online and its potential implications for mental health. Further research with larger sample sizes is warranted to enhance our understanding of these associations and their implications for diagnosis and treatment. The study conducted by van Den Eijnden et al. suggests that excessive but non-disordered use of gaming and social media may not have a significant negative impact on well-being among teenagers aged 12-15 years [9]. However, disordered use of social media and gaming (defined as a pattern of repeated and excessive engagement in activities that significantly impairs an individual's functionality or causes extreme distress; it is characterized by the inability to control behavior despite negative consequences in various spheres of life) was found to be associated with lower social competence, life satisfaction, and psychosocial well-being, particularly among boys [16]. It was also noted that girls exhibited decreased academic performance.

The gender disparity in the impact of disordered use of gaming and social media could be attributed to the different game genres preferred by boys and girls. Boys tend to gravitate towards genres such as real-time strategy, first-person shooter, and sports, which may have a higher addictive potential [9]. On the other hand, girls show a preference for genres like music and dance, cards, and puzzles, which may have a lesser impact in terms of addiction. Another study by Kim et al. explored the relationship between game genres, personality traits, and IGD. This study had a larger sample size (3217) and a more robust design, suggesting that its findings carry more weight [29]. It implies that the specific genre of games played can influence personality traits and the likelihood of developing IGD. These findings highlight the need to consider not only the overall use of gaming and social media but also the specific patterns and genres of use when examining their impact on psychological, social, and academic outcomes in adolescents. Understanding these nuances can inform targeted interventions and preventive measures to address the potential risks associated with disordered use while recognizing that moderate and non-disordered use may not necessarily have detrimental effects on well-being.

General mental health, eating disorders, and suicidality: the study conducted by Kerr et al. aimed to examine the relationship between different forms of online digital media consumption and mental health outcomes in Canadian teenagers aged 12-17 years. The study involved a large sample size of 13,600 participants and focused on various mental health indicators, including eating disorder symptoms, suicidal ideation and attempts, and overall mental health [22,33-34]. The findings indicated that increased use of social media, text messaging, and video messaging was associated with more severe eating disorder symptoms in both boys and girls, as well as lower overall mental health. The use of messaging applications like Snapchat, which allow for image modification and filtering, was found to contribute significantly to body image issues and increased emphasis on appearance and peer comparison [35-36]. The study also revealed gender differences in the associations between online digital media consumption and mental health outcomes. Girls exhibited higher levels of suicidal ideation, while boys showed elevated rates of suicidal attempts, which could be attributed to experiences of cyber-victimization (the experience of being targeted, harassed, or harmed by others through various forms of online communication and technology platforms, such as social media, online forums, email, or messaging apps; it involves acts of aggression, harassment, bullying, or intimidation conducted through digital means. Cyber victimization can take different forms, including cyberbullying, online harassment, stalking, or spreading harmful rumors or content online) [19-20]. However, these associations were no longer significant when accounting for cyber victimization and adequate sleep.

Contrary to common assumptions, internet gaming was found to be associated with favorable mental health outcomes for girls but not boys. This challenges the notion that the games boys play have a negative impact on their aggressive behavior. Another study supported the idea that the gaming genre is related to specific personality traits [29]. The content of online media was highlighted as a crucial factor in understanding its impact on mental health [21,37-38]. Using social media for communication and making plans with friends was found to be a protective factor against suicidality. However, using social media for public posting and attention-seeking behavior was associated with an increased risk of mental health issues. These findings emphasize the importance of considering specific types of online digital media consumption and their content when examining their effects on mental health outcomes. It is essential to understand the nuances and complexities of online media use to develop targeted interventions and strategies for promoting positive mental health in adolescents.

Indeed, the findings of the study conducted by Kerr et al. should be interpreted by taking its limitations into account. One of the limitations mentioned is the timing of mental health symptoms in relation to digital media use. It is possible that teenagers with preexisting mental health issues may turn to social media as a coping mechanism or as a way to seek support. This suggests a bidirectional relationship between mental health and digital media use, where both factors can influence each other. Understanding the chronology of symptoms and the temporal relationship between digital media use and mental health outcomes is important for gaining a deeper understanding of the impact of internet addiction. Future research should aim to incorporate longitudinal designs and consider the temporal sequencing of symptoms to provide a clearer picture of the causal pathways and potential mechanisms involved.

Sleep disturbances: the study conducted by Tereshchenko et al. highlights the potential association between internet addiction and sleep problems among Russian school-aged adolescents (n=4615). The mechanisms underlying this connection are still not fully understood, but several factors have been proposed [13]. One factor is the proximity of electronic devices, such as smartphones, during sleep. Many teenagers admitted to sleeping with their phones nearby, which can disrupt their sleep patterns [39]. Additionally, exposure to screen light, particularly the blue spectrum, may inhibit the release of the hormone melatonin, which controls the sleep-wake cycle [40-41]. Internet-dependent behaviors, such as contemplation before bed, can also contribute to sleep problems. Ruminating about unpleasant experiences, analyzing failures, and feeling incompetent can lead to increased cognitive arousal and make it difficult for individuals to fall asleep. The study found that excessive social media use was associated with shorter sleep duration, longer time to fall asleep (sleep onset latency), and insomnia, particularly during the beginning and middle of the night [13-14]. Disruptions in circadian rhythm, lower sleep quality, and daytime sleepiness were also reported. Notably, the study observed that boys aged 12-14 years with internet gaming addictions had the greatest impact on sleep quality, which can have implications for their academic performance. The findings emphasize the need for further research to better understand and address sleep issues related to internet addiction in adolescents. Providing guidance and support to affected adolescents and their families can help in overcoming and preventing these sleep problems, which can have a significant impact on their overall well-being and academic functioning.

Depression, ADHD, and ASDS: the study conducted by Restrepo et al. examined a sample of children and adolescents (n=564) between the ages of 7-15 years. The researchers investigated the association between PIU and the presence of serious mental health disorders. The study utilized both parental reports (PR) and self-reports (SR) from the students to compare the findings [11]. For participants below the age of 11 years, a trained research assistant read and explained several components of the study before collecting their responses. The analysis revealed significant positive correlations between PIU and depressive disorders (both SR and PR: p=0.01), a combined subtype of ADHD (SR: P=0.01), and ASD (PR only, potentially due to limitations in self-awareness: p<0.001). These associations remained significant even after controlling for factors such as ethnicity, gender, site, single caregiver, age, socioeconomic status, and any other relevant diagnoses.

These findings were further supported by a separate study conducted by Fujita et al., which assessed clinical depression symptoms using the Patient Health Questionnaire-9 (PHQ-9) scale and clinical anxiety in youth aged 10-18 years by using the General Anxiety Disorder-7 (GAD-7) scale [7]. The results indicated a positive correlation between PIU and both depression and anxiety symptoms [7,42]. Additionally, the study observed that school refusal behavior and social withdrawal often overlapped, and these reactions could be considered typical responses to distressing school-related events [43]. In an attempt to temporarily alleviate their stress, children may choose not to attend school to avoid potential sources of anxiety, such as bullying or academic difficulties. According to Fujita et al., children with PIU may experience higher levels of social recognition online compared to offline interactions with the general population. Consequently, they may opt for social isolation and spend more time online. It is important to note that the generalizability of these study findings is limited due to the absence of randomization and a high dropout rate, with nearly half of the patients failing to complete the questionnaire.

One of the investigations carried out by Jeong et al. focused on examining the relationship between depression and IGD among Korean schoolchildren aged 9-12 years. The study particularly emphasized the connection between depression and IGD both before and after the 12-month follow-up of the ICURE study, identifying it as one of the study's most significant findings [2]. The researchers demonstrated that depression and IGD are intertwined and share common neuronal pathways. Aberrant functioning in certain brain areas such as the prefrontal cortex, amygdala, and gyrus was observed in both depression and IGD cases [2,44-45]. Another theory proposed in the study is social displacement (when a person devotes more time to a particular activity, they have less time available to engage in other activities) [46]. Children who spend their entire day playing video games may lack opportunities for socializing with others. This reduced social engagement, which is crucial for children's psychosocial development, can lead to negative emotions and exacerbate depressive symptoms.

According to the study, compared to kids without IGD symptoms at baseline, kids who had IGD symptoms at the start of the trial were more likely to have depressed symptoms at the 12-month follow-up. In contrast, after adjusting for potential confounding variables, children who initially experienced depressive symptoms had a greater chance of experiencing IGD symptoms at the 12-month follow-up compared to children who did not. The data analysis revealed bidirectional associations between the severity of IGD features and the level of depression. Online interactions that attempt to distract individuals from their troubles and depression may contribute to a detrimental cycle that worsens depression [12]. Although not all results from the study were statistically significant, it is recommended to recognize the reciprocal relationship between IGD and depression and how they mutually influence each other. Such understanding could serve as an important initial step in preventing the development of both conditions.

Exacerbation Outcomes

Taking into account the exacerbation of preexisting mental disorders is of paramount importance. A study conducted by Tzang et al. in Taiwan found that 83% of children with IGD also had ADHD [23,47]. Children with ADHD often turn to video games as a means of distraction from their academic work. Moreover, when children with ADHD are exposed to excessive amounts of violent media during gaming sessions, they may exhibit increased hostility. This study, which involved 102 children aged 7-18 years, was conducted during the onset of the coronavirus disease 2019 (COVID-19) pandemic [23]. Researchers utilized structural equation modeling to assess the role of IGD in exacerbating the transition from ADHD to DMDD (persistent irritability, anger, and frequent intense temper outbursts, with at least three severe anger episodes per week lasting for a year, with its onset occurring before the age of 10 years).

According to a theory, children with ADHD may experience impairments in the positive valence system (controls positive motivational settings or contexts, such as reward seeking, consummatory behavior, and reward/habit acquisition) and cognition system, particularly in working memory [23,48]. Children with IGD may exhibit abnormalities in cognitive, positive valence, negative valence (the primary driver of reactions to aversive situations or contexts, such as dread, anxiety, and loss), and social process systems. As a result, children with both IGD and ADHD may display a combination of executive function and motivational salience abnormalities [23,48]. Another theory suggests that adolescents with IGD and untreated ADHD may have a genetic predisposition that exacerbates their symptoms of impulsivity, irritability, and ADHD [23]. IGD may progressively increase the hereditary risk in teenagers with untreated ADHD, leading to the manifestation of more severe symptoms resembling DMDD. The findings of the study revealed a significant association between IGD and poor interpersonal relationships, as well as DMDD-like symptoms in over half of the ADHD youths with IGD, compared to ADHD youths without IGD. This suggests that IGD may play a mediating role in the risk of disruptive mood dysregulation symptoms in children with ADHD. This information highlights the importance of early intervention for children with ADHD and is valuable in informing parents about the potential consequences of untreated ADHD.

Fatal Outcomes

The question of whether playing internet games can lead to death is a topic of controversy. There have been reports of individuals passing away while engaged in video games, particularly after prolonged periods of playing without food, drink, or sleep [49]. There have also been incidents of violence and suicides where video games were cited as the main motivating factor. A study conducted by Kuperczko et al. aimed to identify cases reported in online newspapers, blogs, and video portals where a gamer's death was directly attributed to playing the game (excluding suicides or homicides driven by frustration, anger, impulse, retaliation, or carelessness) [24]. Most of the 24 victims identified were from internet cafes in Southeast Asia. A common characteristic observed was that the games required intense mental focus, and the competitive nature of the games pushed players to continuously engage without taking breaks. Players would become completely absorbed, losing track of time and neglecting basic needs such as thirst, hunger, or rest, which could have fatal consequences. There have been instances where excessive gaming time has been linked to fatal pulmonary embolism and deep vein thrombosis [24]. Acute autonomic dysfunction caused by stress and lack of sleep can also lead to arrhythmias, which can be a contributing factor to death in certain cases. It is likely that many of the victims were suffering from IGD. However, the number of reported cases of fatal gaming incidents appears to be relatively low, significantly fewer than the average occurrence of sudden cardiac death related to common sports activities in the same risk group. It is important to note that this study only collected data from notable cases reported in newspapers and international media, and no comprehensive studies or case reports explaining this pattern have been identified to date. Nevertheless, the severity of these outcomes calls for further investigation and research.

Beneficial Outcomes

While we have discussed the negative effects of excessive use of digital media and video games on mental health, it is important to acknowledge the potential benefits that gaming and digital media use can have for children and adolescents. Some studies suggest that social media can help maintain relationships across boundaries and distances, while video games can provide ways to relax, relieve stress, and improve problem-solving abilities [9,25]. Various studies have identified a correlation between playing video games and experiencing thriving mental health [17,26-27]. These studies have employed Seligman's positive psychology framework, known as the PERMA model (Positive emotion, Engagement, Relationships, Meaning and purpose, and Accomplishment), to investigate how video games and technology can enhance psychological well-being.

Researchers have found that moderate levels of video game play can create an environment that enhances mental health by improving mood, promoting relaxation, and enhancing emotion control. It can also reduce emotional disturbances and stress. Interestingly, the optimal results are associated with moderate playing - not too much or too little. As opposed to highlighting negative effects, these studies have found that playing video games is linked to higher self-esteem in areas such as intelligence, computer skills, and mechanical aptitude. Additionally, Islam et al. found that adolescents who spent an average amount of time playing video games on weekdays or weekends outperformed those who did not play at all in terms of reading and academic performance [15]. Moreover, playing games may contribute to the strengthening of cognitive abilities, further supporting academic growth. However, it can be challenging for parents to effectively regulate their children's and teenagers' screen time to ensure it falls within the recommended timeframe of around two hours per day. This process is not always successful, and finding a balance between the benefits and potential risks of gaming and digital media use is crucial. Parental involvement and guidance play a significant role in fostering healthy and responsible media habits among children and adolescents.

Recommendations

We strongly recommend that parents and educators play an active role in promoting the safe and responsible use of video games and social media among children and adolescents. This approach allows them to fully harness the positive impacts while minimizing any potential negative consequences. Implementing strict laws and guidelines regarding screen time limits and age-appropriate content, along with increased monitoring and supervision, can be effective preventive measures. It is also important to encourage healthy social connections and relationships among young individuals and to be vigilant in recognizing early signs of mental health concerns. Additionally, Geisel et al.'s research has demonstrated the effectiveness of cognitive-behavioral therapy in the prevention and treatment of internet-related disorders. However, further research is needed to better understand and address these issues. By taking proactive measures and staying informed about the potential risks and benefits, parents and educators can contribute to the well-being and healthy development of children and adolescents in the digital age.

Limitations

Our systematic review has several limitations that need to be acknowledged. Firstly, the reliance on self-reported, online, and parental questionnaires for assessment in the majority of the included studies may have introduced certain biases. While this method has its value, it is important to note that many individuals who test positive for psychiatric disorders based on these questionnaires may not meet the diagnostic criteria when evaluated by a psychiatrist. Conversely, there is also a risk of misclassifying true positives as negatives during screening, particularly in populations with a low prevalence of the condition. These limitations raise concerns about the generalizability of our findings and the potential for recall and selection biases. Additionally, the use of cross-sectional data in most studies limits our ability to establish the temporal relationships between gaming, digital media use, and mental illness. Longitudinal studies that follow individuals over time would provide more robust evidence in this regard. Furthermore, our review was constrained by the availability of data published only in English over the past five years. This restriction may have resulted in the exclusion of relevant studies conducted in other languages or older research that could have contributed valuable insights.

To address these limitations, future research should consider incorporating systematic, in-depth psychiatric interviews following the criteria outlined in DSM-5 for evaluating internet-related disorders and IGD. This would provide a more comprehensive and standardized approach to assessing mental health conditions related to gaming and digital media use. Additionally, expanding the scope to include studies beyond children and adolescents and incorporating diverse populations would enhance the generalizability of findings.

## Conclusions

The debate surrounding social networking and online gaming should be approached with nuance, recognizing that the relationship between digital media consumption and mental health is influenced by multiple factors. Future research needs to delve into these factors to gain a comprehensive understanding of this complex relationship. At the same time, it is crucial to address and provide support to manage mental health issues that may arise from the inappropriate use of digital media and online gaming. Families of teenagers who experience negative consequences require assistance and compassion. Interventions should be developed to help parents, teachers, and other caregivers understand the potential benefits and drawbacks of young people's use of digital media. Restoring and maintaining a strong parent-child relationship is also essential, as it may have been strained due to the stress and challenges associated with digital media use. By taking a balanced approach, acknowledging both the positive and negative aspects of digital media consumption, and providing appropriate support and interventions, we can strive for healthier and more responsible use of technology among young individuals while addressing any mental health issues that may arise.
